# Worsened Anxiety and Loneliness Influenced Gaming and Gambling during the COVID-19 Pandemic

**DOI:** 10.3390/jcm12010249

**Published:** 2022-12-29

**Authors:** Mohamed S. Mohamed, Gull Rukh, Helgi B. Schiöth, Sofia Vadlin, Susanne Olofsdotter, Cecilia Åslund, Kent W. Nilsson

**Affiliations:** 1 Center for Clinical Research, Västmanland County Hospital Västerås, Uppsala University, 721 89 Västerås, Sweden; 2 Functional Pharmacology Unit, Department of Surgical Sciences, Uppsala University, 751 24 Uppsala, Sweden; 3 Department of Psychology, Uppsala University, 751 42 Uppsala, Sweden; 4 Department of Public Health and Caring Sciences, Uppsala University, 751 23 Uppsala, Sweden; 5 Division of Public Health Sciences, School of Health, Care and Social Welfare, Mälardalen University, 721 23 Västerås, Sweden

**Keywords:** gaming, problem gambling, anxiety, depression, COVID-19, social isolation

## Abstract

**Aim:** To study the prevalence and patterns of problematic gaming and gambling during the COVID-19 pandemic and the association with psychiatric traits and major types of anxiety categories. **Method:** 1067 young adults participated in both wave 3 (2018) and wave 4 (2021) of the SALVe Cohort. Associations with psychiatric symptoms and anxiety were examined using logistic regression and Chi-square tests. **Results:** Problematic gaming decreased by 1.3 percentage points to 23.2% since the start of the pandemic, while problematic gambling increased by 0.9 percentage points to 6.5% in w4. Average time spent playing video games/day decreased from 2.2 h (w3) to 1.7 h (w4), while increases in gaming activity were associated with worsened feelings of loneliness (*p* = 0.002), depression (*p* < 0.001), and anxiety (*p* < 0.01) during the pandemic. Predictors for problematic gaming at w4 were previous problematic gaming and social anxiety (*p* = < 0.001 and 0.01, respectively). Moreover, previous problem gambling also predicted problem gambling at w4 *p* < 0.001. All anxiety categories were associated with both problematic gaming and gambling when adjusted for age and sex. However, after adjusting for depression and insomnia, social anxiety was associated with problematic gaming (*p* < 0.001), while panic was associated with problem gambling (*p* < 0.001). **Conclusion:** Overall, problematic gaming has decreased since the start of the pandemic, while problem gambling has increased. Worsened feelings of loneliness, depression, and anxiety during the pandemic are associated with increased gaming. Moreover, the association between problematic gaming and gambling and anxiety is independent of depression and sleep problems.

## 1. Introduction

Gaming is a common leisure activity that has been shown to have some beneficial effects in adolescence when used in moderation resulting in stronger social connections and positive academic performance [1,2]. Nevertheless, gaming may constitute a maladaptive coping strategy leading to the development of a gaming disorder [3]. Internet gaming disorder (IGD) was introduced in Section three, “Conditions for further studies”, in the DSM-5 [4]. Subsequently, gaming disorder was included in the World Health Organization (WHO) International Classification of Diseases (ICD-11) in 2019 and was characterized by impaired control over gaming, prioritizing gaming to the degree it takes precedence over other activities, and continuation despite adverse effects [5]. The rationale behind IGD inclusion in the DSM-5 for further studies was the need for studies describing the natural course, the identification of possible genetic/biological factors, and associated comorbidities [4,6,7]. Prior to this, gambling disorder was the only behavioral (non-substance) addiction disorder in the DSM-5 [4,8]. Similarly, gaps in research on gambling disorder included the need for developing preventive programs/efficacy studies, understanding the link with non-gambling games, and the identification of risk factors and comorbidities [9,10,11].

The field of behavioral addictions is relatively new and understanding the relationship between both disorders and psychiatric morbidities is of paramount importance to help establish better screening and intervention strategies. Research in this domain is limited, and the available literature shows that gaming and gambling disorders, albeit with conflicting results, are associated with psychiatric disorders, e.g., depression and anxiety [6,12,13,14,15]. However, recent systematic reviews and a gap analysis have revealed that differences in methodology, lack of longitudinal studies, and the need for clarified models and indexes are the basic limitations to reaching a consensus on the topic [10,11,12,16]. Systematic reviews reporting an association between gaming disorder and mental illness, e.g., depression and anxiety, had low-quality evidence and selective outcome reporting towards positive results [17]. Furthermore, whether psychiatric comorbidities act as a risk factor or a consequence of gaming disorders remains to be clarified [12]. The overlap and co-occurrence between mental illnesses [18,19] further impede our understanding of the relationship between gaming and gambling disorders and psychiatric morbidities.

Moreover, the COVID-19 pandemic has created a mental health crisis with increased social isolation, stress, and loneliness [20]. The WHO issued a warning for mental health services showing a striking 25% increase in the prevalence of anxiety and depression during the first year of the pandemic [21]. These conditions, along with a shift to online gaming/gambling venues due to COVID-19 restrictions, have raised the need for studies to understand the effects of COVID-19 on problem gamblers/gamers, vulnerable groups, and the general population [22,23].

Specifically, studies exploring the association between specific anxiety categories and gaming and gambling disorders are scarce and inconclusive. Studies investigating the relationship with gaming disorders are mostly cross-sectional [13,24,25,26,27] and have a small sample size [13,25,27]. Some studies that have shown an association between specific anxiety disorders (social phobia [24], generalized anxiety disorder ”GAD” [27]) did not include other anxiety disorders nor controlled for co-occurrent morbidities. Likewise, studies on the association between gaming and gambling disorders and specific anxiety disorders are insufficient and have yielded inconsistent results [14,15,28].

In this study, we aim to address these limitations by (a) longitudinally studying the natural course of problematic gaming and gambling since the start of the pandemic in a sample of 1067 young adults over a three-year period; (b) identifying the pattern of gaming and gambling behaviour throughout the COVID-19 pandemic and deciphering traits associated with changes in the pattern; (c) helping to elucidate the link between the symptoms of different anxiety categories as comorbidities and/or risk factors for problem gaming and gambling by systematically controlling for potential confounding factors/comorbidities.

## 2. Materials and Methods

### 2.1. Participants

**Figure 1:**
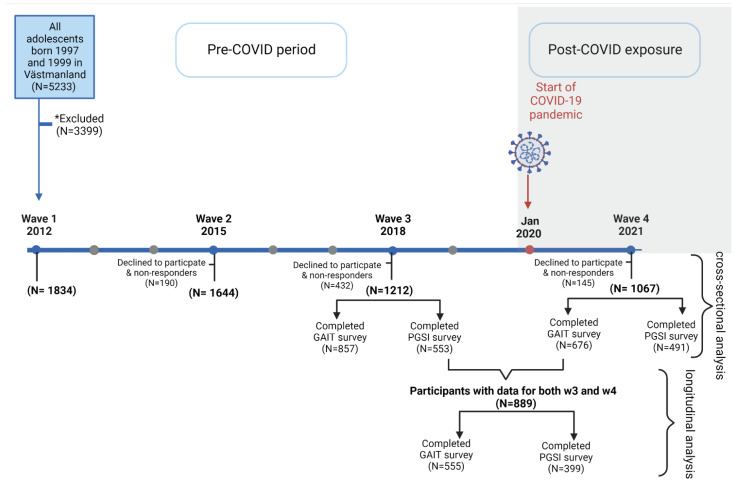
**Flowchart of the study population:** the SALVe cohort study started in 2012 (wave 1). Cross-sectional analysis was performed for wave 4 responders (N = 1067), while longitudinal analysis was performed for those who completed both waves (N = 889). * Excluded due to language difficulties, severe mental disabilities or illness, not living in Västmanland in 2012, declined to participate and non-responders. GAIT = Gaming Addiction Identification Test, PGSI = Problem Gambling Severity Index.

Adolescents born in 1997 and 1999 and living in the county of Västmanland in Sweden were asked to participate in the prospective cohort study “the Survey of Adolescent Life in Västmanland Cohort” (SALVe cohort) that started in 2012 by completing a self-report questionnaire. So far, data at four time points have been collected in the SALVe cohort represented by wave 1 to wave 4 (w4), as shown in Figure 1. In the present study, data from wave 3 (w3), regarded as the pre-COVID-19 period, and from w4, regarded as the post-COVID exposure, were used. Thus, 1067 young adults who completed w4 on October 2021 were included in the cross-sectional analysis, and of these, 889 participants also completed the w3 questionnaire on November 2018 and were included in the longitudinal analysis. Gaming activity and problematic gaming were measured by the Gaming Addiction Identification Test (GAIT). GAIT was completed by 676 participants in w4 and 857 in w3. To assess gambling activity and problem gambling, the Problem Gambling Severity Index (PGSI) was used and was completed by 491 participants in w4 and 553 in w3. For the longitudinal analysis, 555 completed the GAIT survey and 399 completed the PGSI.

### 2.2. Measurements

The GAIT scale was used to assess the average time spent on video games as well as symptoms of gaming addiction in the last 12 months. GAIT is comprised of 15 items with a maximum possible total of 60 points on a scale ranging from 0 = disagree to 4 = Completely agree. The scale has been shown to have high internal consistency and good content validity in identifying gaming addiction [29,30]. Internal consistency was measured by Cronbach’s alpha for this study and was α = 0.883 in w4 and α = 0.890 in w3. GAIT was divided into quartiles for a logistical regression analysis for both w4 and w3 as follows; Q1 = 0 (28.3% w4, 30.6% w3), Q2 = 1–3 (21.7% w4, 23.9% w3), Q3 = 3–8 (26.8% w4, 21.0% w3), Q4 ≥ 9 (23.2% w4, 24.5% w3), with Q1 set as a reference category and Q4 individuals as problematic gamers.

The PGSI scale was used to assess problem gambling. It consists of 9 items with a possible maximum score of 27 points to measure problem gambling severity in the general population. The PGSI has a response scale ranging from “0 = Never” to “3 = almost always”, and a cutoff of ≥3 is commonly used to indicate problem gambling [30]. The PGSI scale showed high internal consistency measured by Cronbach’s alpha and was α = 0.940 in w4 and α = 0.879 in w3.

To evaluate the associations with anxiety categories, the Adult Anxiety Scale (AAS-15) was used. The AAS-15 is a self-rating scale for the measurement of anxiety symptoms in adults [31]. The AAS-15 consists of 15 items to assess symptoms of panic, social anxiety and generalized anxiety. Pilot studies have shown adequate psychometric properties for the AAS-15 [31]. The AAS-15 has a response scale ranging from “0 = Never” to “3 = always”, with a total score of 45 points. Social anxiety, generalized anxiety, and panic subscales had a total score of 15 points. Internal consistency was measured by Cronbach’s alpha, and AAS-15 showed high consistency in both waves (α = 0.933 in w4 and α = 0.928 in w3).

To further look at the effects of the COVID-19 restrictions period, a separate question was added to the GAIT, PGSI, and AAS-15 surveys (Has your gaming/gambling/anxiety changed since the start of the COVID-19 pandemic?) at w4. The question had a response scale from 0 to 3 (0 = Yes, increased, 1 = Yes, decreased, 2 = Unchanged, 3 = Never played/had anxiety before or after the pandemic).

### 2.3. Control Variables

To identify different anxiety categories associated with problematic gaming and gambling, a three-model system for logistic regression analysis was developed to control for age, sex, depression and sleep problems/insomnia. Both depression and insomnia have been shown to be associated with anxiety, problematic gaming, and problem gambling [19,32,33,34,35]. The first model (Model I) was controlled for age and sex. The second model (Model II) was controlled for depression in addition to age and sex, and the third model (Model III) was controlled for Model II and sleep.

Age was coded by year of birth (1997 & 1999), and sex was coded by 1 = male, 2 = female. Depression was measured by the Depression Self-Rating Scale (DSRS). The scale covers the DSM-IV criteria for depression in adolescents and has shown high internal consistency in the previous SALVe waves [36]. Internal consistency for DSRS was measured by Cronbach’s alpha and was α = 0.857 in w4 and α = 0.865 in w3. The Karolinska Sleep Questionnaire (KSQ) was used to assess sleep quality, sleepiness, and symptoms of insomnia with good criterion and construct validity and reliability [37]. The KSQ internal consistency was assessed by Cronbach’s alpha in this study and was α = 0.911 in w4 and α = 0.909 in w3.

### 2.4. Statistical Analysis

The mean, standard deviation, and percentages were calculated for descriptive statistics. Internal consistency was measured by Cronbach’s alpha for GAIT, PGSI, AAS-15, DSRS, and KSQ for both waves. For cross-sectional analysis, logistic regression was performed to study the associations between problematic gaming and anxiety categories in wave 4 using the GAIT quartile scores, AAS-15 subscales for anxiety (generalized anxiety, social anxiety, panic) and adjusted for age, sex, depression, and sleep. Associations between anxiety categories and problem gambling were measured using the PGSI scale with a cutoff ≥ 3 for problem gambling, AAS-15 subscales for anxiety (generalized anxiety, social anxiety, panic), and adjusted for age, sex, depression, and sleep. For longitudinal analysis, logistic regression was performed to predict problematic gaming and problematic gambling in w4 using the GAIT score quartiles for gaming and categorical PGSI for gambling, AAS-15 subscales for anxiety, and adjusted for age, sex, depression, and sleep. To further investigate the association between problem gambling and gaming, GAIT was added to both analyses. Chi-square tests were performed to analyze sex differences and changes associated with increased gaming during the COVID-19 pandemic. The sample size for increased gambling during the pandemic was too small for further analysis. Statistical analysis was performed using the Statistical Package for Social Sciences (version 26), and a *p*-value < 0.05 was considered significant.

## 3. Results

### 3.1. Descriptive Attributes of w4 and w3 Participants

**Figure 2:**
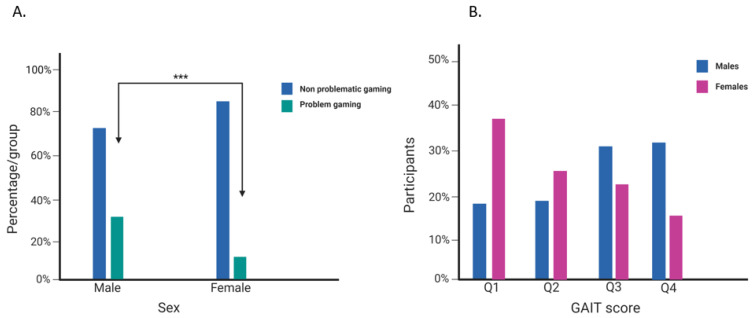
**Sex differences in problematic gaming at w4:** (**A**) on the left, percentage of problematic gaming among males and females is shown. Males are twice more likely (30.4%) to have problem gaming compared to females (15.8%). (**B**) On the right, percentage of GAIT quartile scores among males and females is shown. The majority of males (61.9%) scored in the third and fourth quartiles, while majority of females (61.4%) scored in the first and second quartiles. *** represents statistically significant associations (*p*-value < 0.0001).

**Figure 3:**
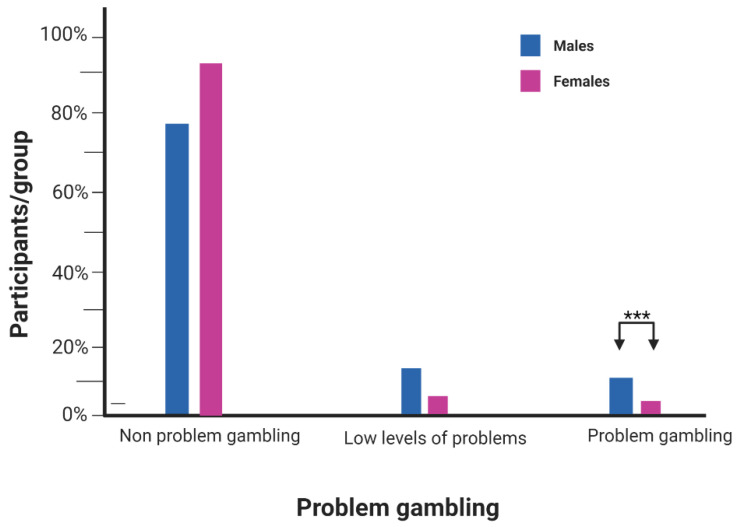
**Sex differences in problem gambling at w4:** male sex (10.9%) is approximately four folds more likely to be problem gamblers compared to females (2.7%). Majority of males (76%) and females (92%) were non-problem gamblers. *** represents statistically significant associations (*p*-value < 0.0001).

The mean age for participants was 22.9 years for w4 and 20.1 years for w3 (Table 1). Females constituted 64.4% of w4 participants and 66.3% in the longitudinal analysis. In the post-COVID-19 exposure group, the male sex was associated with higher GAIT scores and problematic gaming (Figure 2). Males were approximately two folds more likely to be problem gamers (*p* < 0.001) and tended to have a score in the highest quartiles (Q3 = 30.5%, Q4 = 31.7%). In contrast, the majority of females had a score in the lowest quartiles (Q1 = 37.4%, Q2 = 24.1%). Likewise, problem gambling was approximately four folds more likely in male participants compared to females (*p* < 0.001) (Figure 3). Both GAIT and PGSI mean scores slightly increased at w4 compared to the pre-COVID-19 period (GAIT: 5.76 w3 to 5.88 w4; PGSI: 0.51 w3 to 0.61 w4). However, problem gaming decreased during the pandemic by 1.3 percentage points, while problematic gambling increased by 0.9 percentage points. The mean average time spent on gaming per day has also decreased since the start of the pandemic (Table 1).

### 3.2. Changes in Gaming and Gambling Patterns during the COVID-19 Pandemic

Additional questions regarding changes in gaming and gambling behaviour were added to both the GAIT and PGSI scales to assess the effects of the COVID-19 pandemic. Gaming increased in 12.3% of participants, 11.1% reported a decrease, and 38.2% did not change their gaming during the pandemic (Table 2). Regarding gambling behaviour, 1.7% reported an increase, 2.6% reported a decrease, and 30.1% reported no changes in their gambling behaviour during the COVID-19 pandemic.

**Figure 4:**
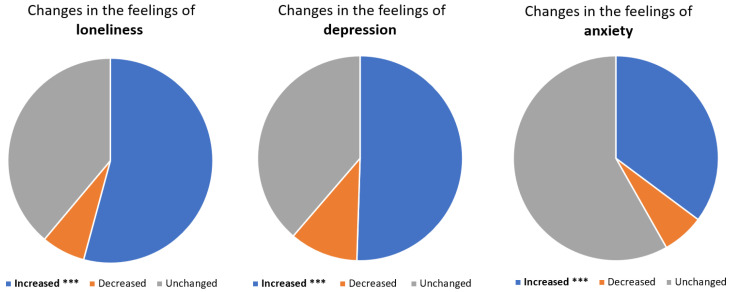
**Changes in the psychological traits among participants with increased gaming activity during the COVID-19 pandemic:** participants who reported an increase in their gaming were more likely to report an increase in feelings of loneliness (54.2%, *p* < 0.001), feelings of depression (50.5%. *p* < 0.001), and anxiety (35.2%, <0.001). *** represents *p*-value < 0.001.

Furthermore, during the pandemic, roughly half of the participants who increased their gaming also reported an increase in feelings of loneliness (54.2%, *p* < 0.001) and depression (50.5%, *p* < 0.001) (Figure 4). Comparatively, in those who decreased their gaming, increased feelings of depression were reported in only 27.8%, while a decreased level of depression was reported in 21.1%. Similarly, increased loneliness was reported in 32.5% of participants who reported less gaming activity during the pandemic.

Additionally, increased feelings of anxiety were reported by 35.2% of those who increased their gaming (*p* < 0.001) compared to 18.5% of those who reported less gaming activity during the COVID-19 pandemic. Finding a way to deal with social loneliness did not have an association with increased gaming during the pandemic *p* = 0.46 (not shown in figures). The small sample size of participants with increased gambling limited further analysis.

### 3.3. Cross-Sectional Association between Psychiatric Traits and Problematic Gaming and Gambling in SALVe Cohort at Wave 4

Logistic regression analysis was performed using the three-model system to identify anxiety categories associated with problematic gaming and gambling. For problem gaming, all anxiety categories had a significant association in the first model (Table 3). After adjusting for depression in Model II, social anxiety and generalized anxiety remained associated with problem gaming. Only social anxiety was associated with problem gaming after adjusting for insomnia (Model III), OR = 1.193, (95% CI 1.117–1.274), *p* < 0.001.

Similarly, problem gambling was associated with all anxiety categories in the first model (Table 4). However, social anxiety was not associated with problem gambling after adjusting for depression. Panic was the only category associated with problem gambling in Model III, OR = 1.224, (95% CI 1.078–1.390), *p* = 0.002.

Logistic regression analysis revealed that young adults with problem gaming had more than a two-fold probability of having gambling problems and problematic gaming remained significantly associated with problematic gambling in all three models (Model III; OR = 2.383, (95% CI 1.221–4.651), *p* = 0.011 (Table 4).

### 3.4. Longitudinal Association between Psychiatric Traits and Problematic Gaming and Gambling in SALVe Cohort at Wave 4

To study the association between psychiatric traits before the COVID-19 pandemic and problem gaming and gambling following the pandemic, data from young adults who participated in both w4 and w3 were used. Logistic regression analysis was performed using the three-model system to investigate predictors of problem gaming and gambling. After adjusting for age, sex, depression, and sleep at w3, social anxiety and previous problem gaming at w3 were predictors for problematic gaming at w4, OR = 1.134, (95% CI 1.061–1.212), *p* < 0.001 and OR = 3.834, (95% CI 2.817–5.220), *p* ≤ 0.001, respectively (Table 5).

On the other hand, panic predicted problem gambling when adjusted for age and sex, OR = 1.273, (95% CI 1.098–1.476), *p* = 0.001. However, after adjusting for depression, none of the anxiety categories predicted problem gambling in w4 (Table 6). Previous gambling problems were the only predictor for problem gambling at w4 in Model III, OR = 3.834, (95% CI 2.817–5.220), *p* < 0.001.

## 4. Discussion

The present study aimed to explore the course of problematic gaming and gambling and the changes in gaming and gambling behaviour during the COVID-19 pandemic. Since the start of the pandemic, the mean average time spent on video games decreased from 2.2 h to 1.7 h in wave 4. Increased gaming activity was reported in 12.3%, while increased gambling was reported in only 1.7% of responders and limited further analysis. Interestingly, young adults who increased their gaming during the pandemic were more likely to report an increase in feelings of loneliness, depression, and anxiety. Moreover, problem gaming was reported in 23.2% of respondents at w4, representing a decrease of 1.3 percentage points since the start of the COVID-19 pandemic. On the other hand, our analysis demonstrated that problematic gambling has increased since the start of the pandemic by 0.9 percentage points and was reported in 6.5% of participants. Notably, participants of the SALVe cohort were only allowed to gamble legally from w3 according to Swedish law (18 years age minimum).

Data from w4 in the SALVe project were collected in the fall of 2021, thus providing insights into the overall impact of the pandemic on behavioural addictions and mental health. The Swedish national COVID-19 strategy differed substantially from other European and Nordic countries [38,39,40]. During the first wave (from March 2020), the Swedish Public Health Agency (Folkhälsomyndigheten) emphasized the role of individual responsibilities and voluntary cooperation. The agency recommended a limit of 50 people for public gatherings with no mandatory restrictions or lockdowns. Following the recommendations, higher education institutions switched to distance learning, and roughly half of the Swedish workforce started working remotely from home [40]. The recommended limit for gatherings was then increased to 300 in October 2020 [38]. Subsequently, the Swedish government approved a pandemic law in January 2021 to limit gatherings and opening hours in public places, e.g., restaurants and bars closed at 8:30 pm until June 2021, when closing time was extended to 10:30 pm. All restrictions were then lifted by the end of September 2021 [40,41]. The adverse psychological impact of COVID-19 in Sweden during the first phase resulted in a surge of depression, anxiety, and insomnia [42]. In addition, the majority of university students reported worsened mental health problems due to campus closure with increased stress, anxiety, and loneliness [43].

Similarly, the associations between increased gaming and increases in feelings of depression, anxiety, and loneliness may be explained via the role of personal distress (due to COVID-19) as a mediator between loneliness, anxiety, and compensatory and problematic gaming [44,45]. Likewise, fear of missing out (FOMO) from social experiences/rewards was shown to mediate the relationship between anxiety and problematic gaming [46]. Compared to the present study, a survey study in Japan showed contradictory results with a 1.5-fold increase in gaming disorders [47]. The study used data only from the early stages of the pandemic (December 2019–July 2020), a period with the most restrictions and social isolation, which might explain the variance as our study used data up to October 2021. Furthermore, previous literature has shown that the natural course of excessive and problematic gaming tends to decline through age [48,49]. Similarly, the present study follow-up from late adolescence age to young adults in w4 may have influenced the decrease in problem gamers in the study. Regarding problem gambling, previous studies investigating problem gambling and COVID-19 have shown varied results, with a reduction in gambling reported in some due to lack of access during the first lockdown phase, while an increase in others has been reported due to mental health issues and financial pressures [50]. Most articles, however, were of cross-sectional design and used data during the first restriction phase of the pandemic, limiting the interpretation of the overall trend [50]. Additionally, sex differences were observed in problematic gaming and gambling, with males two-fold more likely to have problematic gaming and four-fold more likely to be problem gamblers, similar to previous findings [30,51].

Longitudinal analysis showed that pre-pandemic problematic gaming was a strong predictor of problem gaming at w4 (*p* < 0.001). This result supported findings on predictors of IGD in adolescents [30,52]. Gambling problems at w3 were similarly associated with problem gambling (*p* < 0.001). Furthermore, problem gaming showed an association with gambling problems cross-sectionally. Notably, pre-pandemic problematic gaming was not a predictor of gambling problems at w4. The cross-sectional association has been described recently [53] with the rise in online in-game purchasing, such as loot boxes implicated in the association and the transition into problem gambling [54]. Interestingly, these results in a young adult population resembled our findings in previous SALVe cohorts among adolescents [30]. This long follow-up period provides important insights into the natural course and persistence of problematic gaming and gambling throughout age groups.

Furthermore, the present study pursued the association between anxiety categories as comorbidities and/or predictors and problematic gaming and gambling. After controlling in Model III, social anxiety remained strongly associated with problematic gaming (*p* = 1.37 × 10^−7^), while panic was the only anxiety category associated with problem gambling (*p* = 0.002). Our finding demonstrated that problematic gaming is independently associated with social anxiety when controlled for depression and sleep, whereas previous studies did not control for depression and were instead one of the outcomes studied [24,26,27,55]. Depression has also been described, along with anxiety, as a mediator between gaming disorder and insomnia [19]. In contrast, panic was the only anxiety category associated with problematic gambling in Model III. This result was particularly interesting as studies on the association between major anxiety categories and problem gambling are limited. In contrast to our finding, a recent case-control study examining the association between problem gambling and anxiety revealed that social phobia had the strongest association with problem gambling [14]. The study used a different design and did not control for depression and sleep, which might explain the variation. On the other hand, our study is similar to an early nation-representative study utilizing the DSM-IV criteria for pathological gambling in the U.S., that showed panic disorder to have the strongest association with problem gambling [28].

To further elucidate the relationship between anxiety and problem gaming and gambling, data from participants who completed both waves (2018 and 2021) were used to study anxiety symptoms as predictors of problematic gaming and gambling. Comparable to the cross-sectional analysis, social anxiety was the only anxiety category to predict problematic gaming at w4 (*p* < 0.001). This result indicated that social anxiety is not merely a consequence of problematic gaming but may trigger the development of gaming problems. Moreover, it gives strong evidence on the directionality of the association between problematic gaming and anxiety that has previously been shown to be inconclusive with contradictory results [56,57]. Nonetheless, social anxiety has been shown to have an association with escape motives and both positive and negative metacognition about gaming and IGD [58]. In contrast, a comprehensive longitudinal study in Norway found anxiety to be a consequence of pathological and addicted gaming [59]. Regarding problem gambling, panic was the only anxiety category to predict gambling problems at MI, but the association was insignificant after adjusting for depression (Model II). The results are similar to a U.S. survey study that showed anxiety is not associated with problem gambling after accounting for other predictors [1].

The present study has several strengths and limitations. Firstly, the strength of our study lies in the mixed-method design with longitudinal analysis and the use of repeated measurements, enabling comparisons. Secondly, the additional questions on the effects of COVID-19 on participants, coupled with the longer follow-up period, enabled an understanding of the role of the pandemic through its different stages by reducing the acute influence of the first months of the pandemic. Thirdly, a three-model system was used to control for co-occurrent and overlapping comorbidities to decipher the strength of the associations between anxiety categories and the role of anxiety as a cause and/or a consequence of problematic gaming and gambling. The limitations of our study are the reliance on self-reported symptoms, albeit the measurements were repeated in both waves. The unique approach to the COVID-19 pandemic in Sweden with minimal restrictions further limited comparisons with countries that had strict lockdowns and mandatory restrictions. In addition, the low number of participants that completed the PGSI survey and the percentage of problem gamblers limited our analysis further. Moreover, we used GAIT score quartiles to categorize problem gamers which may have influenced the results.

## 5. Conclusions

The present study offers important insights into the course of problematic gaming and gambling and the association with psychiatric traits since the start of the COVID-19 pandemic. Problematic gaming and average time spent on games decreased while problematic gambling increased during the pandemic. The study shows that participants who increased their gaming were more likely to report increased feelings of loneliness, depression, and anxiety. Moreover, problematic gaming was associated with problem gambling cross-sectionally but was not a predictor of future gambling problems. Future studies investigating the interplay and overlap between gaming and gambling behaviours are warranted, especially given the rise of online in-game purchasing of loot boxes.

Furthermore, the present study demonstrated that anxiety symptoms associated with problematic gaming and gambling are independent of depression and sleep disturbances. The surge in anxiety disorders since the start of the COVID-19 pandemic is thus proving troublesome for the management of gaming and gambling problems. Screening and preventive plans should address anxiety disorders for vulnerable groups, and measurements to reduce anxiety may help decrease the impact of gaming and gambling disorders. Future research, including clinical samples and objective measurements (e.g., online gaming records), may help in understanding the relationship between anxiety, depression, and gaming and gambling disorders.

## Figures and Tables

**Table 1:** Descriptive statistics for measurements in the third and fourth wave of the SALVe Cohort.

Characteristics		Wave 3 (N = 1212)	Wave 4 (N = 1067)	* Longitudinal Analysis (N = 889)
**Scale Mean (SD)**				
Age		20.10 (1.09)	22.9 (1.04)	22.9 (1.04)
Sleep/insomnia (KSQ)		26.25 (14.25)	26.64 (14.29)	26.63 (14.34)
Deprssion (DSRS)		3.09 (2.84)	3.36 (2.86)	3.33 (2.83)
Gaming (GAIT)		5.76 (7.69)	5.88 (7.80)	5.70 (7.50)
Average time spent in video game/day (hours)		2.18(2.13)	1.71(2.049)	1.66 (2.01)
Gambling (PGSI)		0.51 (1.79)	0.61 (2.57)	0.36 (1.23)
**Categorical N (%)**				
Sex	Male	458 (37.8%)	380 (35.6%)	300 (33.7%)
	Female	754 (62.2%)	687 (64.4%)	589 (66.3%)
Gaming (GAIT)	No problem	647 (75.5%)	519 (76.8%)	430 (77.5%)
	Problem gaming	210 (24.5%)	157 (23.2%)	125 (22.5%)
Gambling (PGSI)	No problem	457 (82.6%)	415 (84.5%)	344 (86.2%)
	Low level of problems	65 (11.8%)	44 (9%)	35 (8.8%)
	Problem gambling	31 (5.6%)	32 (6.5%)	20 (5.0%)

Abbreviations: KSQ, Karolinska Sleep Questionnaire; DSRS, Depression Self Rating Scale; GAIT, Gaming Addiction Identification Test; PGSI, Problem Gambling Severity Index. * Wave 4 data from participants that completed both wave 3 and wave 4.

**Table 2:** Changes in gaming and gambling behaviour during the COVID-19 pandemic.

Characteristic	Increased during the Pandemic N (%)	Decreased during the Pandemic N (%)	Unchanged N (%)	Never Played before nor after the Pandemic N (%)
Gaming	131 (12.3%)	118 (11.1%)	408 (38.2%)	410 (38.4%)
Gambling	18 (1.7%)	28 (2.6%)	321 (30.1%)	700 (65.6%)

**Table 3:** Logistic regression model of the cross-sectional association between anxiety categories and problematic gaming.

Anxiety Trait	Model I *				Model II **				Model III ***			
	Odds Ratio	95% Confidence Interval		*p*-Value	Odds Ratio	95% Confidence Interval		*p*-Value	Odds Ratio	95% Confidence Interval		*p*-Value
		Lower CI	Upper CI			Lower CI	Upper CI			Lower CI	Upper CI	
Social anxiety	1.26	1.18	1.33	**<0.001**	1.20	1.13	1.28	**<0.001**	1.19	1.12	1.27	**<0.001**
Generalized anxiety	1.17	1.11	1.24	**<0.001**	1.08	1.01	1.16	**<0.05**	1.06	0.99	1.14	0.090
Panic	1.22	1.12	1.32	**<0.001**	1.08	0.98	1.19	0.110	1.05	0.95	1.16	0.308

Abbreviations: CI, confidence interval; *p*-values in bold represent statistically significant associations. * Adjusted for age and sex, ** Adjusted for age, sex and depression (DSRS), *** Adjusted for age, sex, depression (DSRS), and insomnia (KSQ).

**Table 4:** Logistic regression model of the cross-sectional association between psychiatric traits and problem gambling.

Trait	Model I *				Model II **				Model III ***			
	Odds Ratio	95% Confidence Interval		*p*-Value	Odds Ratio	95% Confidence Interval		*p*-Value	Odds Ratio	95% Confidence Interval		*p*-Value
		Lower CI	Upper CI			Lower CI	Upper CI			Lower CI	Upper CI	
Social anxiety	1.18	1.08	1.28	**<0.001**	1.09	0.99	1.21	0.061	1.07	0.97	1.18	0.18
Generalized anxiety	1.22	1.11	1.33	**<0.001**	1.13	1.02	1.26	**<0.05**	1.08	0.97	1.22	0.17
Panic	1.36	1.22	1.51	**<0.001**	1.29	1.14	1.45	**<0.001**	1.23	1.08	1.39	**<0.01**
Problem gaming (GAIT)	2.87	1.48	5.54	**<0.01**	2.36	1.22	4.57	**<0.05**	2.38	1.22	4.65	**<0.05**

Abbreviations: CI, confidence interval; GAIT, Gaming Addiction Identification Test. *p*-values in bold represent statistically significant associations. * Adjusted for age and sex, ** Adjusted for age, sex and depression (DSRS), *** Adjusted for age, sex, depression (DSRS), and insomnia (KSQ).

**Table 5:** Longitudinal association between psychiatric traits at w3 and problem gaming in SALVe cohort at Wave 4.

Traits	Model I *				Model II **				Model III ***			
	Odds Ratio	95% Confidence Interval		*p*-Value	Odds Ratio	95% Confidence Interval		*p*-Value	Odds Ratio	95% Confidence Interval		*p*-Value
		Lower CI	Upper CI			Lower CI	Upper CI			Lower CI	Upper CI	
Social anxiety	1.17	1.10	1.25	**<0.001**	1.14	1.07	1.22	**<0.001**	1.13	1.06	1.21	**<0.001**
Generalized anxiety	1.10	1.04	1.17	**<0.01**	1.05	0.98	1.13	0.18	1.03	0.96	1.11	0.45
Panic	1.19	1.08	1.32	**<0.001**	1.11	0.99	1.24	0.07	1.08	0.96	1.21	0.21
Problem gaming (GAIT)	4.02	2.95	5.46	**<0.001**	3.91	2.87	5.32	**<0.001**	3.83	2.82	5.22	**<0.001**

Abbreviations: CI, confidence interval; GAIT, Gaming Addiction Identification Test. *p*-values in bold represent statistically significant associations. * Adjusted for age and sex, ** Adjusted for age, sex, and depression (DSRS), *** Adjusted for age, sex, depression (DSRS), and insomnia (KSQ).

**Table 6:** Longitudinal association between psychiatric traits at w3 and problem gambling in SALVe cohort at Wave 4.

Traits	Model I *				Model II **				Model III ***			
	Odds Ratio	95% Confidence Interval		*p*-Value	Odds Ratio	95% Confidence Interval		*p*-Value	Odds Ratio	95% Confidence Interval		*p*-Value
		Lower CI	Upper CI			Lower CI	Upper CI			Lower CI	Upper CI	
Social anxiety	1.13	1.02	1.25	0.02	1.06	0.95	1.24	0.27	1.05	0.93	1.18	0.42
Generalized anxiety	1.09	0.98	1.21	0.10	0.97	0.86	1.10	0.71	0.95	0.83	1.08	0.43
Panic	1.27	1.18	1.48	**0.001**	1.15	0.96	1.37	0.13	1.12	0.93	1.35	0.24
Problem gaming (GAIT)	1.68	1.02	2.72	0.41	1.55	0.97	2.51	0.70	1.53	0.94	2.48	0.09
Problem gambling (PGSI)	6.92	3.07	15.61	**<0.001**	5.94	2.58	13.66	**<0.001**	6.05	2.61	14.06	**<0.001**

Abbreviations: CI, confidence interval; GAIT, Gaming Addiction Identification Test; PGSI, Problem Gambling Severity Index. *p*-values in bold represent statistically significant associations. * Adjusted for age and sex, ** Adjusted for age, sex and depression (DSRS), *** Adjusted for age, sex, depression (DSRS), and insomnia (KSQ).

## Data Availability

The data presented in this study are available on request from the corresponding author on reasonable request. The data are not publicly available due to privacy and ethical restrictions.
